# Impact of COVID-19 pandemic on prescription stimulant use among children and youth: a population-based study

**DOI:** 10.1007/s00787-023-02346-x

**Published:** 2024-01-05

**Authors:** Tony Antoniou, Kathleen Pajer, William Gardner, Melanie Penner, Yona Lunsky, Daniel McCormack, Mina Tadrous, Muhammad Mamdani, Peter Gozdyra, David N. Juurlink, Tara Gomes

**Affiliations:** 1 Li Ka Shing Knowledge Institute, St. Michael’s Hospital, Toronto, ON Canada; 2 ICES, Toronto, ON Canada; 3 Department of Family and Community Medicine, University of Toronto, Toronto, ON Canada; 4 Department of Family and Community Medicine, St. Michael’s Hospital, Toronto, ON Canada; 5 Children’s Hospital of Eastern Ontario Research Institute, Ottawa, ON Canada; 6 Department of Psychiatry, University of Ottawa, Ottawa, ON Canada; 7 School of Epidemiology and Public Health, University of Ottawa, Ottawa, ON Canada; 8 Autism Research Centre, Bloorview Research Institute, Holland Bloorview Kids Rehabilitation Hospital, Toronto, Canada; 9 Department of Pediatrics, University of Toronto, Toronto, ON Canada; 10 Azrieli Adult Neurodevelopmental Centre, Centre for Addiction and Mental Health, Toronto, Canada; 11 Department of Psychiatry, University of Toronto, Toronto, ON Canada; 12 Leslie Dan Faculty of Pharmacy, University of Toronto, Toronto, ON Canada; 13 Li Ka Shing Centre for Healthcare Analytics Research & Training, Unity Health Toronto, Toronto, ON Canada; 14 Temerty Faculty of Medicine, University of Toronto, Toronto, ON Canada; 15 Institute of Health Policy, Management, and Evaluation, University of Toronto, Toronto, ON Canada; 16 Department of Medicine, University of Toronto, Toronto, ON Canada

**Keywords:** Child, Adolescent, Central nervous system stimulants, Prescriptions/statistics & numerical data, Policy

## Abstract

COVID-19 associated public health measures and school closures exacerbated symptoms in some children and youth with attention-deficit hyperactivity disorder (ADHD). Less well understood is how the pandemic influenced patterns of prescription stimulant use. We conducted a population-based study of stimulant dispensing to children and youth ≤ 24 years old between January 1, 2013, and June 30, 2022. We used structural break analyses to identify the pandemic month(s) when changes in the dispensing of stimulants occurred. We used interrupted time series models to quantify changes in dispensing following the structural break and compare observed and expected stimulant use. Our main outcome was the change in the monthly rate of stimulant use per 100,000 children and youth. Following an initial immediate decline of 60.1 individuals per 100,000 (95% confidence interval [CI] − 99.0 to − 21.2), the monthly rate of stimulant dispensing increased by 11.8 individuals per 100,000 (95% CI 10.0–13.6), with the greatest increases in trend observed among females, individuals in the highest income neighbourhoods, and those aged 20 to 24. Observed rates were between 3.9% (95% CI 1.7–6.2%) and 36.9% (95% CI 34.3–39.5%) higher than predicted among females from June 2020 onward and between 7.1% (95% CI 4.2–10.0%) and 50.7% (95% CI 47.0–54.4%) higher than expected among individuals aged 20–24 from May 2020 onward. Additional research is needed to ascertain the appropriateness of stimulant use and to develop strategies supporting children and youth with ADHD during future periods of long-term stressors.

**Supplementary Information:**

The online version contains supplementary material available at 10.1007/s00787-023-02346-x.

## Introduction

Public health restrictions imposed during the COVID-19 pandemic exacted a considerable toll on the mental health of children and youth [1, 2]. Those with ADHD were especially vulnerable to the loss of daily routines associated with school closures and other public health measures, with several studies finding increases in the severity of ADHD symptoms, anxiety, increased screen time, and negative mood [3–10]. Specifically, a study of more than 600 youth with ADHD and individually-matched comparators found that those with ADHD were more likely to exhibit sleeping difficulties, fear and negative emotions related to the risk of family illness, trouble with remote learning, rule-breaking behavior related to COVID-19 restrictions and family conflict [11]. Moreover, youth with ADHD were less prepared for the next school year and less responsive to protective environmental variables’ mitigating effects, such as school engagement and parental monitoring [11]. Further, a study of 118 youth with ADHD and a comparison group of 110 individuals without ADHD found that those with ADHD had fewer routines, higher negative affect and more difficulties with remote learning than those without ADHD [12]. These challenges were amplified among adolescents with ADHD requiring school accommodations, with nearly one-third of these adolescents’ parents indicating that remote learning was very challenging, compared with 18% of parents of adolescents with ADHD and no learning plan and 4% of parents with neither ADHD nor pre-existing learning difficulties [12].Remote learning may have therefore exacerbated academic difficulties already experienced by youth with ADHD [13, 14].

However, comparatively little research has examined whether pandemic-associated challenges for children and youth with ADHD impacted prescription stimulant use. This is important, as stimulants are the mainstay of pharmacotherapy for children and youth with ADHD, and the rapid transition to virtual care may have disrupted ongoing ADHD care and decreased access to stimulant pharmacotherapy [15, 16]. In Ontario specifically, physician billing codes for virtual assessments were introduced in March 2020 [17], with virtual visits comprising the majority of outpatient mental health visits for children and adolescents during the ensuing months of the COVID-19 pandemic [18]. There were no restrictions placed on stimulant prescribing during this period. In order to facilitate treatment continuity following the transition to virtual care, Health Canada implemented changes to the Controlled Drugs and Substances Act that permitted pharmacists to verbally accept, adapt, extend and transfer prescriptions for patients using controlled substances such as stimulants [19]. Although the proportion of pharmacist submitted stimulant claims increased 21.5 fold following the amendment, the absolute number of stimulant claims submitted by pharmacists remained low, increasing from an average of 0.02% of all submitted claims per month during the pre-pandemic period to 0.42% of all submitted claims per month between March 22, 2020 and December 19, 2021 [19].

In contrast to concerns about decreased stimulant access, the loss of school-based and non-pharmacological services during the pandemic may have promoted increased stimulant use and amplified pre-pandemic trends of increased stimulant use among children and youth described in other studies [20, 21] This is important, as stimulants have been associated with adverse effects such as decreased appetite, weight loss, sleep difficulties, and irritability [22, 23]. Stimulants have also been associated with growth suppression and rare adverse effects such as psychosis and priapism [22–26]. Cardiovascular adverse effects are generally limited to modest increases in blood pressure and heart rate [27]. The risk of cardiovascular adverse effects may be heightened with concomitant use of antipsychotics or fluoroquinolone antibiotics [28, 29]. Furthermore, the increased use of stimulants among females of child-bearing age and during pregnancy has raised concerns about the potential for adverse neonatal outcomes following in utero exposure to these drugs [30–32]. Although some studies have found associations between in utero stimulant exposure and cardiac malformations, gastroschisis, omphalocele, and transverse limb deficiency, the absolute risks appear small [33, 34]. Moreover, antenatal exposure to stimulants was not associated with neurodevelopmental or growth outcomes in a large cohort study [35], and a systematic review of eight cohort studies found no convincing evidence associating prenatal prescription stimulant exposure with clinically significant adverse effects [36]. Another potential concern with prescription stimulants is the potential for misuse among young adults. Past research has found that non-medical use of these drugs increased from 3.7% to 4.5% of surveyed university students in Canada between 2013 and 2016 [37, 38]. Reports of serious adverse events with non-medical stimulant use, such as critical care hospitalizations, emergency department visits for stimulant toxicity and death, highlight the risks associated with this practice [39].

However, despite concerns about interruptions in the continuity of care and the potential risks of stimulants, available studies examining the impact of COVID on stimulant use in children and youth are hampered by limited follow-up and little clarity regarding whether changes in stimulant use varied by sex, age and socioeconomic status [40–42]. Accordingly, we studied the impact of the COVID-19 pandemic on prescription stimulant use among the entire population of individuals aged 0–24 in Ontario, home to approximately 40% of Canadian children and youth [43].

## Methods

### Setting

We conducted a population-based study of all Ontario residents aged 0–24 years between January 1, 2013, and June 30, 2022. As in other jurisdictions, Ontario students experienced several cycles of school openings and closures during the pandemic (see supplemental appendix for timeline), and experienced the most frequent fully-remote school closures in Canada, totalling approximately 220 days during the pandemic [44, 45].

### Data sources

We used Ontario’s administrative health databases which were linked using unique encrypted identifiers and analyzed at ICES. These databases are valid and reliable for routine sociodemographic data, physician billing claims and primary diagnoses, and undergo routine rigorous quality checks for completeness and consistency by the Data Quality and Information Management team at ICES [46, 47]. We identified stimulant prescriptions using the Narcotics Monitoring System database, which contains comprehensive records of prescriptions for stimulants dispensed from community pharmacies in Ontario, regardless of payer. We used the ICES Corporate Provider Database to determine prescriber specialty and the Registered Persons Database, a registry of all individuals eligible for the publicly-funded Ontario Health Insurance Plan, to ascertain demographic characteristics for all children and youth dispensed stimulants over the study period. The use of data in this project was authorized under Sect. 45 of Ontario’s Personal Health Information Protection Act, which does not require review by a Research Ethics Board.

### Study population and outcomes

For each month of the study period, we defined our study population as all Ontario residents aged 24 and younger who were alive on the first day of the month. Our primary outcome was the monthly rate of stimulant use per 100,000 children and youth, defined as the number of individuals dispensed a stimulant (i.e., amphetamine, dextroamphetamine, lisdexamfetamine, methylphenidate; supplemental Table 1) in each month divided by the population of children and youth aged 0–24 for that period. To ascertain whether the characteristics of children and youth changed following the COVID-19 pandemic, we compared demographic characteristics and prescriber type (i.e., general practitioner, developmental pediatrician and/or pediatric psychiatrist, pediatrician, other) for individuals receiving stimulants during the pre-pandemic (January 2013–March 2020) and post-pandemic (April 2020–June 2022) periods.

**Table 1:** Demographic characteristics of individuals aged 0 to 24 dispensed a stimulant, January 2013 to June 2022

Variable^a^	Entire Study Period (January 1, 2013–June 30, 2022)	Pre-COVID (January 1, 2013–March 31, 2020)	Post-COVID (Apr 1, 2020–Jun 30, 2022)	Standardized Difference Period 1-Period 2
Number of individuals	308,583	241,794	176,562	
Age (median, IQR)	13 (8–18)	13 (8–18)	15 (10–20)	0.29
0–4	5,353 (1.7%)	4,299 (1.8%)	1,271 (0.7%)	0.10
5–9	96,062 (31.1%)	77,584 (32.1%)	37,086 (21.0%)	0.25
10–14	72,400 (23.5%)	60,151 (24.9%)	47,827 (27.1%)	0.05
15–19	74,362 (24.1%)	57,258 (23.7%)	46,075 (26.1%)	0.06
20–24	60,406 (19.6%)	42,502 (17.6%)	44,303 (25.1%)	0.18
Sex				
Female, No. (%)	112,138 (36.3%)	78,677 (32.5%)	69,866 (39.6%)	0.15
Male, No. (%)	196,445 (63.7%)	163,117 (67.5%)	106,696 (60.4%)	0.15
Income quintile				
1 (lowest)	57,153 (18.5%)	45,949 (19.0%)	30,827 (17.5%)	0.04
2	54,896 (17.8%)	43,403 (18.0%)	30,819 (17.5%)	0.01
3	57,750 (18.7%)	45,149 (18.7%)	32,796 (18.6%)	0.00
4	64,340 (20.9%)	50,022 (20.7%)	37,235 (21.1%)	0.01
5	74,444 (24.1%)	57,271 (23.7%)	44,885 (25.4%)	0.04
Residence				
Urban	275,812 (89.4%)	215,820 (89.3%)	157,754 (89.3%)	0.00
Rural	32,771 (10.6%)	25,974 (10.7%)	18,808 (10.7%)	0.00
Prescriber Type				
General Practitioner	124,772 (40.4%)	93,955 (38.9%)	80,410 (45.5%)	0.14
Pediatrician	120,825 (39.2%)	99,573 (41.2%)	63,319 (35.9%)	0.11
Psychiatrist	49,368 (16.0%)	38,421 (15.9%)	23,731 (13.4%)	0.07
Other	13,618 (4.4%)	9,845 (4.1%)	9,102 (5.2%)	0.05
Average days’ supply of prescription (mean, SD)	29.73 ± 18.71	30.68 ± 19.87	31.87 ± 18.44	0.06
Days’ supply category				
1 to 7	26,202 (8.5%)	20,037 (8.3%)	10,333 (5.9%)	0.10
8 to 14	38,595 (12.5%)	29,177 (12.1%)	13,372 (7.6%)	0.15
15 to 29	54,151 (17.5%)	41,787 (17.3%)	33,465 (19.0%)	0.04
> 30	189,625 (61.5%)	150,783 (62.4%)	119,392 (67.6%)	0.11
Stimulant Type				
Amphetamine	27,187 (8.8%)	23,189 (9.6%)	14,304 (8.1%)	0.05
Dextroamphetamine	4,592 (1.5%)	4,198 (1.7%)	1,818 (1.0%)	0.06
Lisdexamfetamine	80,271 (26.0%)	55,709 (23.0%)	62,262 (35.3%)	0.27
Methylphenidate	196,533 (63.7%)	158,698 (65.6%)	98,178 (55.6%)	0.21

### Statistical analyses

We used standardized differences (SD) to compare demographic characteristics between individuals receiving a stimulant during the pre- and post-pandemic periods, with differences greater than 0.1 considered meaningful [48].

We used several approaches to explore whether stimulant dispensing to children and youth changed during the pandemic. First, rather than assuming that changes in stimulant dispensing occurred immediately following the imposition of public health restrictions and school closures in March 2020, we used structural break analyses to test for shifts in the intercept and/or slope of the time series, following seasonal adjustment to account for the lower use of stimulants in the summer months and 15% trimming of the dataset [49, 50]. This approach also allowed us to determine whether other breaks in the time series were associated with events unrelated to the pandemic that would need to be accounted for in subsequent modelling. Next, we determined the crude relative percent changes in stimulant dispensing from the month prior to the structural break to the month following the structural break and to June 2022, the end of our study period.

Because the relative percent change does not account for prior trends, temporal correlation and seasonality, we used interrupted time series analyses to quantify the immediate step change and change in monthly stimulant dispensing trend per 100,000 individuals aged 0–24 years following the structural break(s) [51, 52]. Specifically, we used a dummy variable to denote the timing of the structural break(s), an indicator for time to account for the underlying temporal trend in the data and an interaction term between time and the dummy variable representing the structural break to estimate the change in stimulant dispensing trend following the structural break. Our models also included dummy variables for month to account for seasonality, and a variable denoting implementation of a publicly-funded pharmacare program known as OHIP + that covered the prescription costs of all individuals aged 24 and under beginning in January 2018 [53]. We also calculated expected stimulant dispensing rates for the period following the structural break in the absence of COVID-19 using data between January 2013 and the month preceding the structural break, with time, month, and the indicator for OHIP + as model predictors. We then determined the relative percent changes between the observed and predicted stimulant dispensing rates and estimated associated 95% confidence intervals using the Poisson distribution. To explore heterogeneity in the impact of the pandemic, we stratified all analyses by sex, age category (5–9 years, 10–14 years, 15–19 years, 20–24 years), neighbourhood income quintile and urban versus rural residence, defined on the first day of the month of interest. We tested for autocorrelation to a maximum of 12 lags using the Cumby-Huizinga test for autocorrelation and estimated all models using Newey-West standard errors to account for autocorrelation up to 12 lags and heteroscedasticity [54, 55]. All analyses were completed using Stata version 17.0 (StataCorp LLC, College Station, TX, USA), R Studio, and EViews 12.

## Results

**Fig. 1:**
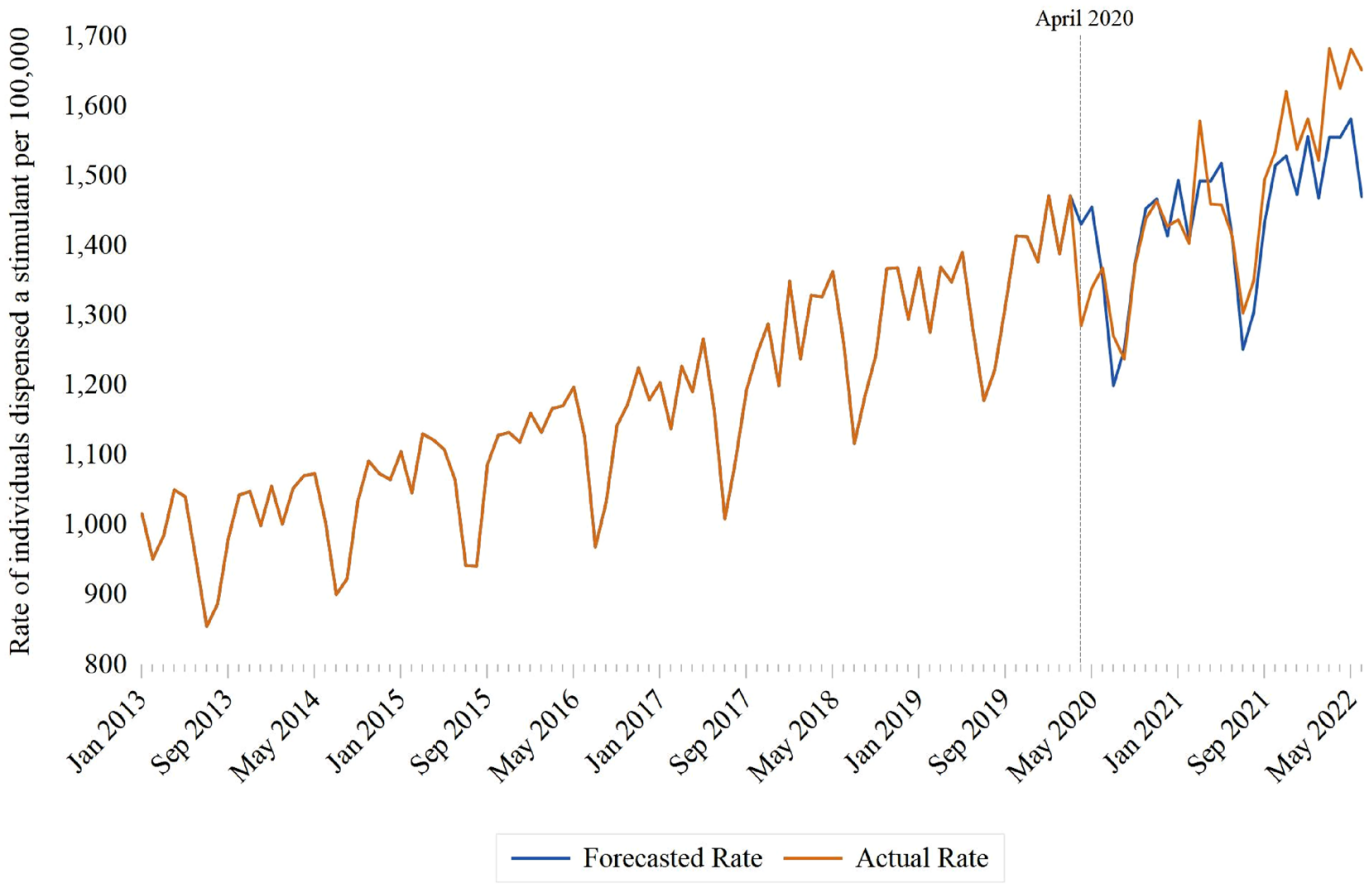
Impact of COVID-19 (April 2020) on monthly rates of stimulant dispensing among Ontario residents between the ages of 0 and 24, January 2013 to June 2022

During our nearly 10-year study period, 308,583 individuals aged 24 or younger were dispensed a stimulant. The rate of stimulant dispensing among children and youth increased 62.6% (95% confidence interval [CI] 60.6–64.5%) between January 2013 (1015.8 per 100,000 individuals) and June 2022 (1651.2 per 100,000 individuals). Most stimulant-treated children and youth were male (n = 196,445; 63.7%), and the median age was 13 years (interquartile range: 8–18 years) (Table 1). Individuals between the ages of 0 and 4 comprised less than 2% of children and youth dispensed a stimulant during the study period (Table 1, supplemental Fig. 1). Compared with the pre-pandemic period, there was an increase in the proportion of stimulant recipients that were female (32.5% vs. 39.6%; SD = 0.15) and those aged 20–24 years (17.6% vs. 25.1%; SD = 0.18). Conversely, there was a decrease in the proportion of stimulant recipients that were male (67.5% vs. 60.4%; SD = 0.15), aged 0–4 years (1.8% vs. 0.7%; SD = 0.15) and aged 5–9 years (32.1% vs. 21.0%; SD = 0.25) (Table 1). Changes in prescriber type were also observed following the pandemic, with an increase in the proportion of stimulant prescriptions written by general practitioners (38.9 vs. 45.5%; SD = 0.14) and a corresponding decline in the share of prescriptions written by pediatricians (41.2 vs. 35.9%; SD = 0.11) (Table 1). Methylphenidate was the most commonly dispensed stimulant during the study period (Table 1). However, methylphenidate accounted for a decreased proportion of stimulant prescriptions in the post-pandemic period relative to the pre-pandemic period (55.6% vs. 65.6%; SD = 0.21). Conversely, lisdexamfetamine accounted for an increased proportion of stimulant prescriptions dispensed in the post- relative to the pre-pandemic period (35.3% vs. 23.0%; SD = 0.27). Stimulant dispensing generally followed a seasonal pattern, with higher use during the school months (Fig. 1) and less cyclicity among those aged 20 to 24 relative to younger children and youth (Fig. 3).

### Stimulant dispensing rate level change immediately following COVID-19

Structural break analyses identified endogenous breaks in January 2018, the month of OHIP + implementation, and April 2020, the month following the declaration of a public health emergency and school closures. We observed a modest relative percent decrease in stimulant dispensing to children and youth immediately following the structural break, with rates decreasing 9.0% (95% CI − 10.0% to − 7.9%) between March 2020 and May 2020 (1470.8 vs. 1338.8 per 100,000 population, respectively) (Table 2). In analyses stratified by age, there was an immediate increase in stimulant dispensing among individuals aged 20–24 (1118.3 vs. 1288.7 individuals per 100,000 population), corresponding to a relative percent increase of 15.2% (95% CI 12.2–18.4%) (Table 2). Conversely, stimulant dispensing declined in other age strata and remained below pre-pandemic levels for children aged 5–9 throughout our post-pandemic follow-up period (Fig. 3; supplemental Table 12).

**Table 2:** Changes in stimulant dispensing following COVID-19 pandemic

	COVID Structural Break(s)	Rates and relative percent changes, relative to structural break					Interrupted Time Series Analysis	
	Structural break(s)	Rate per 100,000, in month preceding break	Rate per 100,000 in month following break	Relative percent change (95% CI), month preceding break to month after	Rate per 100,000, June 2022	Relative per cent change, month preceding break to June 2022	Step change in dispensing rate per 100,000, first month following break	Monthly change in dispensing rate trend per 100,000 post-break
Overall	April 2020	1470.8	1338.8	−9.0% (−10.0% to −7.9%)	1651.2	12.3% (11.0% to 13.5%)	−60.1 (−99.0 to -21.2)	11.8 (10.0 to 13.6)
Sex								
Female	April 2020	946.9	880.2	−7.0% (−9.0% to −5.1%)	1293.5	36.6% (34.0% to 39.2%)	−9.4 (−44.7 to 26.0)	16.9 (16.2 to 17.7)
Male	April 2020	1968.4	1773. 9	−9.9% (−11.1% to −8.6%)	1990.3	1.1% (−0.27% to 2.5%)	−108.8 (−156.6 to −61.0)	6.9 (4.1 to 9.7)
Age								
5 to 9	April 2020	1691.4	1519.0	−11.9% (−14.1% to −9.8%)	1473.8	−12.9% (−15.0% to −10.7%)	−188.2 (−231.5 to −144.9)	−3.3 (−5.0 to −1.6)
10 to 14	April 2020	2482.9	2289.8	−7.8% (−9.6% to -6.0%)	2555.7	2.9% (0.96% to 4.9%)	−91.3 (−164.5 to −18.0)	10.2 (6.9 to 13.4)
15 to 19	April 2020	1684.3	1547.0	−8.2% (−10.4% to −5.9%)	1982.4	17.7% (15.0% to 20.4%)	−95.5 (−137.5 to −53.5)	17.8 (16.2 to 19.4)
20 to 24	May 2020	1118.3	1288.7	15.2% (12.2% to 18.4%)	1841.9	64.7% (60.6% to 68.8%)	91.1 (35.9 to 146.3)	25.7 (23.3 to 28.0)
Income Quintile								
1 (lowest)	April 2020	1438.4	1304.0	−9.3% (−11.8% to −6.9%)	1580.7	9.9% (7.1% to 12.7%)	−92.0 (−118.1 to −66.0)	9.3 (7.6 to 10.9)
2	April 2020	1444.6	1335.9	−7.5% (−10.0% to −5.0%)	1612.8	11.6% (8.7% to 14.6%)	−68.7 (−95.9 to −41.6)	11.0 (9.5 to 12.5)
3	April 2020	1361.7	1254.0	−7.9% (−10.4% to −5.4%)	1533.2	12.6% (9.7% to 15.5%)	−50.4 (−85.4 to −15.4)	10.9 (9.4 to 12.5)
4	April 2020	1451.8	1307.5	−9.9% (−12.2% to −7.6%)	1623.1	11.8% (9.1% to 14.5%)	−44.4 (−100.3 to 11.5)	12.1 (10.4 to 13.8)
5	April 2020	1665.5	1507.5	−9.5% (−11.6% to −7.3%)	1916.1	15.0% (12.4% to 17.6%)	−37.8 (−115.6 to 39.8)	15.5 (12.8 to 18.1)
Rural vs. urban residence								
Rural	April 2020	1832.9	1711.1	−6.6% (−9.8% to −3.5%)	2027.9	10.6% (7.1% to 14.2%)	−44.5 (−111.4 to 22.4)	11.7 (9.6 to 13.8)
Urban	April 2020	1436.2	1303.7	−9.2% (−10.4% to −8.1%)	1615.6	12.5% (11.2% to 13.8%)	−62.5 (−98.0 to −26.9)	11.8 (10.0 to 13.6)

Following interrupted time series modelling, there was an immediate decline in stimulant dispensing of 60.1 per 100,000 (95% CI − 99.0 to − 21.2) associated with the April 2020 structural break. (Fig. 1) (Table 2). In stratified analyses, the largest declines were observed among males (− 108.8 per 100,000; 95% CI − 156.6 to − 61.0) and individuals between the ages of 5 and 9 (− 188.2 per 100,000; 95% CI − 231.5 to − 144.9) (Table 2). In addition, a socioeconomic gradient was observed, with the largest decline occurring among residents of low-income neighbourhoods (− 92.0 per 100,000; 95% CI − 118.1 to − 66.0). Conversely, the level change in stimulant dispensing rates was least pronounced among individuals in the highest-income neighbourhoods (− 37.8 per 100,000 individuals; 95% CI − 115.6–39.8). In contrast to other strata, an immediate increase in stimulant dispensing was observed among individuals 20–24 years of age (91.1 per 100,000; 95% CI 35.9–146.3) (Table 2).

### Change in stimulant dispensing rate trends following COVID-19

**Fig. 2:**
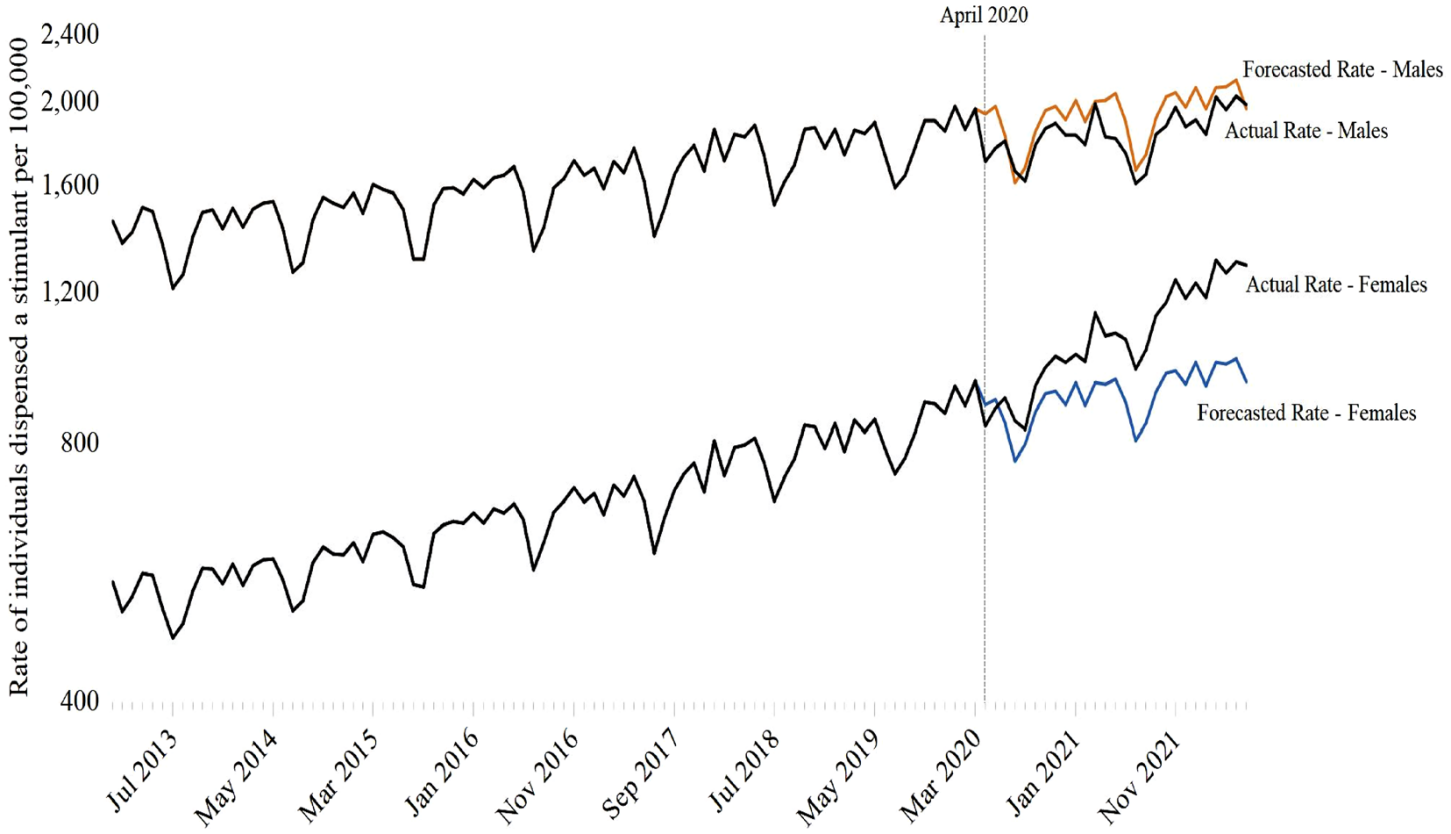
Impact of COVID-19 on monthly rates of stimulant dispensing among Ontario residents between the ages of 0 and 24, January 2013 to June 2022, stratified by sex

**Fig. 3:**
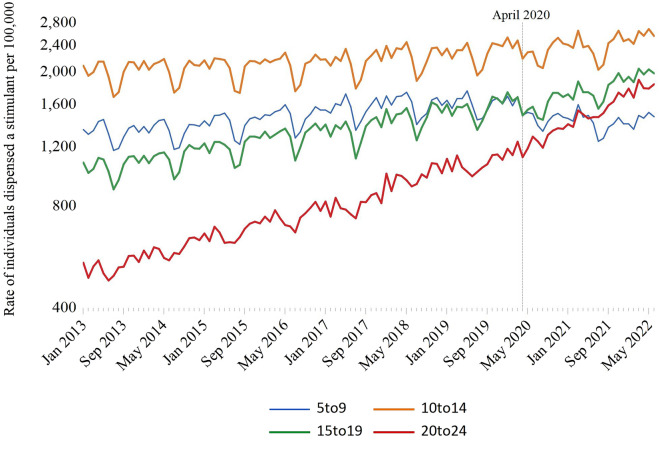
Impact of COVID-19 on monthly rates of stimulant dispensing among Ontario residents between the ages of 0 and 24, January 2013 to June 2022, stratified by age

Stimulant dispensing to children and youth increased in the months following the April 2020 structural break, increasing 12.3% (95 CI 11.0–13.5%) between the month preceding the structural break (i.e., March 2020) and June 2022 (1470.8 vs. 1651.2 individuals per 100,000 population) (Table 2). In stratified analyses, the largest increases were observed among females (946.9 vs. 1293.5 individuals per 100,000) (Fig. 2) and individuals aged 20–24 (1118.3 vs. 1841.9 per 100,000) (Fig. 3), with relative percent increases in stimulant dispensing of 36.6% (34.0–39.2%) and 64.7% (60.6–68.8%), respectively (Table 2).

Interrupted time series models estimated that the monthly rate of stimulant dispensing increased by 11.8 individuals per 100,000 (95% CI 10.0–13.6) after April 2020, with the largest changes in monthly trend observed among females (16.9 individuals per 100,000; 95% CI 16.2–17.7) and individuals aged 20–24 (25.7 individuals per 100,000; 95% CI 23.3–28.0) (Table 2, Figs. 2 and 3). In terms of socioeconomic status, the monthly rate of stimulant dispensing increased most among children and youth living in the highest income neighbourhoods (15.5 individuals per 100,000; 95% CI 12.8–18.1) and least among residents of the lowest income neighbourhoods (9.3 individuals per 100,000; 95% CI 7.6–10.9) (Table 2). In contrast to other strata, monthly stimulant use continued to decline after April 2020 in children aged 5–9 years (− 3.3 individuals per 100,000; 95% CI − 5.0 to − 1.6) (Table 2, Fig. 3).

### Comparison of actual and predicted stimulant dispensing rates following the COVID-19 pandemic

Overall, the observed mean rate of stimulant dispensing between April 2020 and June 2022 was slightly higher than predicted (1464.3 vs. 1440.6 individuals per 100,000), with a relative percent difference of 1.6% (95% CI 0.45–2.8%). However, there was variability in the pattern of change observed, with generally lower than expected rates through May 2021 and observed rates consistently exceeding predicted rates from June 2021 onward (Supplemental Appendix). Although this pattern was similar among most strata of children and youth, observed rates were between 3.9% (95% CI 1.7–6.2%) and 36.9% (95% CI 34.3–39.5%) higher than predicted among females from June 2020 onward (Supplemental Appendix). In addition, youth between the ages of 20 and 24 had stimulant dispensing rates that were between 7.1% (95% CI (4.2–10.0%) and 50.7% (95% CI 47.0–54.4%) higher than expected from May 2020 onward (Supplemental Appendix). Conversely, stimulant dispensing to children aged 5–9 years remained below predicted values at all points during the pandemic (Supplemental Appendix).

## Discussion

In our population-based study, we observed a large increase in stimulant dispensing to children and youth over the study period. This increase may in part reflect the expansion in diagnostic criteria for ADHD in the Diagnostic and Statistical Manual of Mental Disorders (DSM-5), published in May 2013 [24]. With respect to COVID-19, we observed an initial decrease in stimulant dispensing to Ontario children and youth in the early period of the pandemic, followed by a rapid return to pre-pandemic rates in the ensuring months and greater than expected use from June 2021 onward. A similar pattern was observed in a study summarizing stimulant dispensing trends in 47 countries, with greater than expected use beginning in the second quarter of 2021 following a decline early in the pandemic [42]. Our work builds upon these findings by demonstrating considerable heterogeneity in the patterns of stimulant use during the pandemic, with larger post-pandemic increases in stimulant use observed among females, youth between the ages of 20 and 24 and children and youth living in the highest income neighbourhoods. These findings correspond with the changing demographics of stimulant use in the post-pandemic period, with a greater proportion of children and youth receiving stimulants being female and older. Our finding of increased lisdexamfetamine dispensing during the COVID period may reflect trends in stimulant prescribing favouring long-acting formulations and a perception of less misuse potential with this drug [23, 56].

The initial decrease in stimulant use around April 2020 aligns with earlier research demonstrating lower than expected outpatient mental health service use in Ontario children and youth at this point in the pandemic and a lack of severe problem behaviours among youth with ADHD early in the course of the pandemic [10, 18]. In addition, it is possible that this decline reflects parental decisions to discontinue stimulant medication in their children while schools were closed or during remote learning. This assertion may explain the lower than expected stimulant use among children aged 5–9 years, as elementary schools were closed for a large portion of our study period. Conversely, the lack of decline and greater than expected stimulant use during the pandemic among those aged 20–24 could reflect the ability of these individuals to access stimulants without parental involvement. However, a concern with the increased use of stimulants in this specific population is the potential for non-medical misuse of these drugs. A 2020 systematic review summarizing studies of stimulant diversion and misuse found that these practices were most commonly undertaken to enhance academic performance among young adults between the ages of 18 and 25 [39]. Because of the potential for serious adverse effects with the non-medical use of stimulants, additional research is required to ascertain whether stimulant diversion and misuse increased during the pandemic.

Our finding of a greater post-pandemic increase in stimulant use among females relative to males is consistent with studies from other jurisdictions. Specifically, an Australian study of individuals aged 18 years and under found higher than expected stimulant dispensing among females than males [57]. Similarly, a United States study found a substantial increase in stimulant dispensing among females of all age groups in 2020 and 2021, with decreased stimulant dispensing observed among males aged 19 years and younger [58]. Another United States study found that females comprised a greater proportion of first ADHD diagnoses in the pandemic compared to pre-pandemic years [59]. Reasons for sex-based differences in stimulant use following the pandemic are unknown. Because females may be more likely than males to present with the inattentive phenotype of ADHD [60], one possibility is that the transition to virtual learning rendered such symptoms more readily detectable by parents tasked with helping children navigate this new learning paradigm. Similarly, the stressors and disruptions associated with the pandemic may have undermined previously described compensatory behaviours undertaken by females with ADHD to mask symptoms [61].

We also observed differences in stimulant dispensing according to socioeconomic status, with a smaller immediate decline in use followed by a greater increase in the ensuing months among children in the highest relative to the lowest-income neighbourhoods. Other research has described similar trends, with individuals from high-income neighbourhoods returning to pre-pandemic rates of ADHD-related care during the pandemic, while those from the lowest-income neighbourhoods experienced continued disruptions in care [59]. One possible reason for these differences relates to socioeconomic disparities in the ability to work from home, favouring individuals from high-income neighbourhoods [62]. For these families, working from home could have facilitated greater recognition of student challenges with remote learning and subsequent access to stimulant pharmacotherapy to improve performance. Moreover, the transition to virtual health care may have exacerbated inequities in accessing ADHD-related care, with higher-income families having greater access to the resources required to schedule and attend virtual appointments [63]. In addition, a study from the United States found that adolescents from low-income families were significantly more likely than adolescents from high-income families to receive no remote or online learning and significantly less likely to engage in class meetings online [12]. Although it is unknown if a similar pattern occurred in Ontario, less engagement with school may also account for findings of lower stimulant use in lower-income children during the pandemic. Finally, one study found differences in post-COVID symptom manifestation according to socioeconomic status, which may have prompted increased medical attention and treatment, with adolescents from higher-income families being more likely to display opposition and defiant symptoms than those from low-income families [5].

Strengths of our study include complete stimulant claims data for all children and youth in Ontario and greater than two years of post-pandemic follow-up, facilitating a long-term evaluation of patterns in stimulant use during this period. However, our study has some limitations. First, we could not ascertain the appropriateness of stimulant use. Second, our databases do not include information regarding individual income, race, or ethnicity, important determinants of prescription stimulant use in other studies which merit additional research[64, 65]. Third, we could not determine the reasons for differential changes in stimulant use in our study population. Further qualitative and quantitative research is needed to understand these changes and inform programming and policy. Fourth, we did not longitudinally examine whether COVID-19 changed the dose or duration of stimulant. Finally, our study was conducted in a single Canadian province, potentially limiting the generalizability of our findings.

In summary, we found increased stimulant dispensing to children and youth during the pandemic, particularly among females, individuals aged 20–24 and those in the highest-income neighbourhoods. Furthermore, these increases have been sustained during the pandemic. Additional research is needed to ascertain the appropriateness of stimulant use as a response to challenges with virtual learning and the disruption in routines associated with the pandemic and to develop strategies supporting healthy coping behaviours during future periods of long-term stressors during which abrupt disruptions in daily living may occur.

### Supplementary Information

Below is the link to the electronic supplementary material.

Supplementary file 1: (DOCX 197 KB)

## Data Availability

The data set from this study is held securely in coded form at ICES. While data sharing agreements prohibit ICES from making the data set publicly available, access may be granted to those who meet pre-specified criteria for confidential access, available at www.ices.on.ca/DAS. The full data set creation plan and underlying analytic code are available from the authors upon request, understanding that the programs may rely upon coding templates or macros unique to ICES.
