# Supplementary material for: Impact of COVID-19 pandemic on prescription stimulant use among children and youth: a population-based study
Source: Eur Child Adolesc Psychiatry. 2024 Jan 5;33(8):2669–80. doi: 10.1007/s00787-023-02346-x (PMC11272743; doi:10.1007/s00787-023-02346-x)

**Timeline of Pandemic-Associated School Closures in Ontario, Canada**

| **Month** | **Timeline of School Closures and Reopenings** |
| --- | --- |
| 2020-03-14 | Schools closed |
| 2020-03-12 | First post-secondary school closure in province |
| 2020-09-08 | Public schools opened for 1st day of in-person learning; masks required for all staff and grade 4 to 12 students |
| 2021-01-04 | Public and private elementary and secondary school students moved to teacher-led remote learning (7 public health regions) |
| 2021-01-04 | Public and private secondary school students moved to teacher-led remote learning (27 public health regions) |
| 2021-01-12 | Mask use mandated indoors for grades 1 to 3, and outdoors for all students, with expanded testing and screening protocols |
| 2021-01-25 | Elementary/secondary schools resumed in-person learning with testing, asymptomatic screening and mask mandate for grades 1 to 3 (7 regions) |
| 2021-02-01 | Elementary and secondary schools resumed in-person learning (4 additional public health units) |
| 2021-02-08 | Elementary and secondary schools resumed in-person learning (13 additional public health units) |
| 2021-02-16 | Elementary and secondary schools resumed in-person learning in all 3 remaining public health units (Peel, Toronto and York Region) |
| 2021-04-19 | Public and private elementary and secondary schools moved to remote learning |
| 2021-09-07 | Schools open for the 2021–2022 school year; option of returning to in-person learning for full school day or synchronous remote learning |
| 2022-01-05 | All public and private school students resumed virtual learning, in-person return delayed |
| 2022-01-17 | Students returned to in-person learning |
| 2022-02-10 | Extra-curricular activities are allowed again in schools; students are required to wear a mask but may temporarily remove their mask for activities |

**Supplemental Table 1: Stimulants included in study and corresponding Anatomical Therapeutic Chemical (ATC) Classification**

| **Stimulant Name** | **ATC code** |
| --- | --- |
| Amphetamine | N06BA01 |
| Dextroamphetamine | N06BA02 |
| Lisdexamphetamine | N06BA12 |
| Methylphenidate | N06BA04 |

**Supplemental Table 2: Projected and Actual Stimulant Dispensing in Children and Youth, April 2020 to June 2022**

| **Month** | **Projected Rate of stimulant dispensing in absence of COVID pandemic (individuals per 100,000)** | **Actual Rate of stimulant dispensing during COVID pandemic (individuals per 100,000)** | **Relative percent change, actual versus projected stimulant dispensing (95% confidence interval** |
| --- | --- | --- | --- |
| April 2020 | 1430.6 | 1285.4 | -10.1% (-11.2% to -9.1%) |
| May 2020 | 1455.4 | 1338.8 | -8.0% (-9.1% to -6.9%) |
| June 2020 | 1353.4 | 1368.1 | 1.1% (-0.1% to 2.3%) |
| July 2020 | 1199.4 | 1270.4 | 5.9% (4.6% to 7.2%) |
| August 2020 | 1250.9 | 1236.8 | -1.1% (-2.3% to 0.09%) |
| September 2020 | 1374.6 | 1371.6 | -0.22% (-1.4% to 0.95%) |
| October 2020 | 1452.6 | 1438.2 | -1.0% (-2.1% to 0.13%) |
| November 2020 | 1467.0 | 1464.1 | -0.19% (-1.3% to 0.94%) |
| December 2020 | 1413.6 | 1427.2 | 0.96% (-0.2% to 2.1%) |
| January 2021 | 1493.6 | 1436.9 | -3.8% (-4.9% to -2.7%) |
| February 2022 | 1408.6 | 1403.4 | -0.36% (-1.5% to 0.79%) |
| March 2021 | 1492.3 | 1578.5 | 5.8% (4.6% to 7.0%) |
| April 2021 | 1492.5 | 1459.8 | -2.2% (-3.3% to -1.1%) |
| May 2021 | 1518.3 | 1458.3 | -3.9% (-5.0% to -2.9%) |
| June 2021 | 1411.6 | 1414.0 | 0.17% (-1.0% to 1.3%) |
| July 2021 | 1250.8 | 1303.2 | 4.2% (2.9% to 5.4%) |
| August 2021 | 1304.3 | 1349.7 | 3.5% (2.3% to 4.7%) |
| September 2021 | 1433.1 | 1494.0 | 4.2% (3.1% to 5.4%) |
| October 2021 | 1514.2 | 1534.3 | 1.3% (0.20% to 2.5%) |
| November 2021 | 1528.9 | 1621.3 | 6.0% (4.9% to 7.2%) |
| December 2021 | 1473.1 | 1538.5 | 4.4% (3.3% to 5.6%) |
| January 2022 | 1556.3 | 1581.3 | 1.6% (0.49% to 2.7%) |
| February 2022 | 1467.5 | 1521.6 | 3.7% (2.5% to 4.9%) |
| March 2022 | 1554.5 | 1682.7 | 8.2% (7.1% to 9.4%) |
| April 2022 | 1554.5 | 1625.3 | 4.6% (3.4% to 5.7%) |
| May 2022 | 1581.1 | 1681.1 | 6.3% (5.2% to 7.5%) |
| June 2022 | 1469.8 | 1651.2 | 12.3% (11.1% to 13.6%) |

**Supplemental Table 3: Projected and Actual Stimulant Dispensing in Males 0 to 24 years of age, April 2020 to June 2022**

| **Month** | **Projected Rate of stimulant dispensing in absence of COVID pandemic (individuals per 100,000)** | **Actual Rate of stimulant dispensing during COVID pandemic (individuals per 100,000)** | **Relative percent change, actual versus projected stimulant dispensing (95% confidence interval** |
| --- | --- | --- | --- |
| April 2020 | 1944.9 | 1708.3 | -12.2% (-13.4% to -10.9%) |
| May 2020 | 1981.2 | 1773.9 | -10.5% (-11.7% to -9.2%) |
| June 2020 | 1834.1 | 1807.2 | -1.5% (-2.9% to -0.06%) |
| July 2020 | 1613.8 | 1668.6 | 3.4% (1.9% to 4.9%) |
| August 2020 | 1680.6 | 1621.4 | -3.5% (-5.0% to -2.1%) |
| September 2020 | 1853.0 | 1786.4 | -3.6% (-5.0% to -2.2%) |
| October 2020 | 1961.9 | 1870.6 | -4.7% (-6.0% to -3.3%) |
| November 2020 | 1983.9 | 1892.9 | -4.6% (-5.9% to -3.3%) |
| December 2020 | 1910.9 | 1836.3 | -3.9% (-5.2% to -2.6%) |
| January 2021 | 2015.4 | 1833.5 | -9.0% (-10.3% to -7.8%) |
| February 2022 | 1903.4 | 1787.5 | -6.1% (-7.4% to -4.8%) |
| March 2021 | 2012.4 | 1996.2 | -0.8% (-2.1% to 0.54%) |
| April 2021 | 2016.3 | 1828.3 | -9.3% (-10.6% to -8.1%) |
| May 2021 | 2053.7 | 1818.7 | -11.4% (-12.7% to -10.2%) |
| June 2021 | 1901.0 | 1749.3 | -8.0% (-9.3% to -6.7%) |
| July 2021 | 1672.6 | 1611.6 | -3.6% (-5.1% to -2.2%) |
| August 2021 | 1741.6 | 1652.7 | -5.1% (-6.5% to -3.7%) |
| September 2021 | 1920.0 | 1839.7 | -4.2% (-5.5% to -2.8%) |
| October 2021 | 2032.6 | 1880.2 | -7.5% (-8.8% to -6.2%) |
| November 2021 | 2055.2 | 1978.4 | -3.7% (-5.0% to -2.4%) |
| December 2021 | 1979.4 | 1875.3 | -5.3% (-6.6% to -3.9%) |
| January 2022 | 2087.4 | 1911.6 | -8.4% (-9.7% to -7.2%) |
| February 2022 | 1971.2 | 1839.0 | -6.7% (-8.0% to -5.4%) |
| March 2022 | 2083.9 | 2033.9 | -2.4% (-3.7% to -1.1%) |
| April 2022 | 2087.7 | 1964.0 | -5.9% (-7.2% to -4.7%) |
| May 2022 | 2126.3 | 2036.6 | -4.2% (-5.5% to -2.9%) |
| June 2022 | 1968.0 | 1990.3 | 1.1% (-0.26% to 2.5%) |

**Supplemental Table 4: Projected and Actual Stimulant Dispensing in Females 0 to 24 years of age, April 2020 to June 2022**

| **Month** | **Projected Rate of stimulant dispensing in absence of COVID pandemic (individuals per 100,000)** | **Actual Rate of stimulant dispensing during COVID pandemic (individuals per 100,000)** | **Relative percent change, actual versus projected stimulant dispensing (95% confidence interval** |
| --- | --- | --- | --- |
| April 2020 | 888.2 | 839.6 | -5.5% (-7.5% to -3.5%) |
| May 2020 | 900.7 | 880.2 | -2.3% (-4.3% to -0.23%) |
| June 2020 | 847.0 | 905.4 | 6.9% (4.6% to 9.1%) |
| July 2020 | 764.4 | 850.6 | 11.3% (8.9% to 13.7%) |
| August 2020 | 799.9 | 831.4 | 3.9% (1.7% to 6.2%) |
| September 2020 | 871.6 | 934.5 | 7.2% (5.0% to 9.4%) |
| October 2020 | 916.4 | 982.2 | 7.2% (5.0% to 9.3%) |
| November 2020 | 922.1 | 1011.8 | 9.7% (7.5% to 11.9%) |
| December 2020 | 889.7 | 995.7 | 11.9% (9.6% to 14.2%) |
| January 2021 | 944.1 | 1018.4 | 7.9% (5.7% to 10.0%) |
| February 2022 | 886.6 | 998.0 | 12.6% (10.3% to 14.9%) |
| March 2021 | 944.0 | 1137.8 | 20.5% (18.2% to 22.9%) |
| April 2021 | 939.9 | 1070.7 | 13.9% (11.7% to 16.1%) |
| May 2021 | 952.9 | 1077.8 | 13.1% (10.9% to 15.3%) |
| June 2021 | 895.9 | 1059.9 | 18.3% (15.9% to 20.7%) |
| July 2021 | 808.3 | 977.6 | 21.0% (18.4% to 23.5%) |
| August 2021 | 845.6 | 1029.9 | 21.8% (19.3% to 24.3%) |
| September 2021 | 921.1 | 1129.2 | 22.6% (20.2% to 25.0%) |
| October 2021 | 968.3 | 1169.4 | 20.8% (18.5% to 23.1%) |
| November 2021 | 974.0 | 1244.6 | 27.8% (25.4% to 30.2%) |
| December 2021 | 939.6 | 1183.0 | 25.9% (23.5% to 28.3%) |
| January 2022 | 996.8 | 1232.7 | 23.7% (21.4% to 26.0%) |
| February 2022 | 935.9 | 1186.7 | 26.8% (24.3% to 29.2%) |
| March 2022 | 996.2 | 1312.1 | 31.7% (29.3% to 34.2%) |
| April 2022 | 991.7 | 1267.9 | 27.8% (25.4% to 30.2%) |
| May 2022 | 1005.2 | 1306.1 | 29.9% (27.5% to 32.4%) |
| June 2022 | 944.8 | 1293.5 | 36.9% (34.3% to 39.5%) |

**Supplemental Table 5: Projected and Actual Stimulant Dispensing in Urban Residents 0 to 24 years of age, April 2020 to June 2022**

| **Month** | **Projected Rate of stimulant dispensing in absence of COVID pandemic (individuals per 100,000)** | **Actual Rate of stimulant dispensing during COVID pandemic (individuals per 100,000)** | **Relative percent change, actual versus projected stimulant dispensing (95% confidence interval** |
| --- | --- | --- | --- |
| April 2020 | 1401.2 | 1251.2 | -10.7% (-11.8% to -9.6%) |
| May 2020 | 1422.5 | 1303.7 | -8.4% (-9.5% to -7.2%) |
| June 2020 | 1321.5 | 1331.8 | 0.78% (-0.49% to 2.0%) |
| July 2020 | 1167.3 | 1237.5 | 6.0% (4.6% to 7.4%) |
| August 2020 | 1217.5 | 1202.6 | -1.2% (-2.5% to 0.07%) |
| September 2020 | 1343.5 | 1339.9 | -0.27% (-1.5% to 0.97%) |
| October 2020 | 1421.2 | 1405.1 | -1.1% (-2.3% to 0.06%) |
| November 2020 | 1436.9 | 1431.3 | -0.38% (-1.6% to 0.82%) |
| December 2020 | 1381.2 | 1390.8 | 0.69% (-0.54% to 1.9%) |
| January 2021 | 1463.7 | 1403.3 | -4.1% (-5.3% to -3.0%) |
| February 2022 | 1381.3 | 1373.9 | -0.53% (-1.8% to 0.69%) |
| March 2021 | 1462.4 | 1542.8 | 5.5% (4.3% to 6.7%) |
| April 2021 | 1462.8 | 1427.2 | -2.4% (-3.6% to -1.3%) |
| May 2021 | 1484.8 | 1426.0 | -4.0% (-5.1% to -2.8%) |
| June 2021 | 1379.2 | 1381.3 | 0.15% (-1.1% to 1.4%) |
| July 2021 | 1218.1 | 1266.3 | 4.0% (2.6% to 5.3%) |
| August 2021 | 1270.2 | 1316.7 | 3.7% (2.3% to 5.0%) |
| September 2021 | 1401.5 | 1460.9 | 4.2% (3.0% to 5.5%) |
| October 2021 | 1482.4 | 1501.9 | 1.3% (0.12% to 2.5%) |
| November 2021 | 1498.5 | 1587.9 | 6.0% (4.7% to 7.2%) |
| December 2021 | 1440.2 | 1502.6 | 4.3% (3.1% to 5.6%) |
| January 2022 | 1526.0 | 1548.7 | 1.5% (0.30% to 2.7%) |
| February 2022 | 1439.8 | 1492.3 | 3.6% (2.4% to 4.9%) |
| March 2022 | 1524.2 | 1647.1 | 8.1% (6.8% to 9.3%) |
| April 2022 | 1524.4 | 1594.0 | 4.6% (3.3% to 5.8%) |
| May 2022 | 1547.2 | 1645.4 | 6.4% (5.1% to 7.6%) |
| June 2022 | 1436.9 | 1615.6 | 12.4% (11.1% to 13.8%) |

**Supplemental Table 6: Projected and Actual Stimulant Dispensing in Rural Residents 0 to 24 years of age, April 2020 to June 2022**

| **Month** | **Projected Rate of stimulant dispensing in absence of COVID pandemic (individuals per 100,000)** | **Actual Rate of stimulant dispensing during COVID pandemic (individuals per 100,000)** | **Relative percent change, actual versus projected stimulant dispensing (95% confidence interval** |
| --- | --- | --- | --- |
| April 2020 | 1764.8 | 1647.1 | -6.7% (-9.8% to -3.5%) |
| May 2020 | 1820.8 | 1711.1 | -6.0% (-9.2% to -2.9%) |
| June 2020 | 1703.6 | 1752.4 | 2.9% (-0.61% to 6.3%) |
| July 2020 | 1545.4 | 1620.5 | 4.9% (1.2% to 8.6%) |
| August 2020 | 1610.1 | 1598.1 | -0.73% (-4.2% to 2.7%) |
| September 2020 | 1717.7 | 1713.3 | -0.25% (-3.6% to 3.1%) |
| October 2020 | 1803.2 | 1795.9 | -0.40% (-3.7% to 2.9%) |
| November 2020 | 1804.9 | 1818.7 | 0.77% (-2.6% to 4.1%) |
| December 2020 | 1770.0 | 1815.2 | 2.6% (-0.85% to 5.9%) |
| January 2021 | 1844.0 | 1790.8 | -2.9% (-6.1% to 0.30%) |
| February 2022 | 1729.0 | 1718. | -0.63% (-4.0% to 2.7%) |
| March 2021 | 1839.3 | 1955.5 | 6.3% (2.9% to 9.7%) |
| April 2021 | 1838.7 | 1804.5 | -1.9% (-5.1% to 1.3%) |
| May 2021 | 1896.8 | 1800.7 | -5.1% (-8.1% to -2.0%) |
| June 2021 | 1774.5 | 1757.5 | -0.96% (-4.2% to 2.3%) |
| July 2021 | 1609.4 | 1685.9 | 4.8% (1.2% to 8.4%) |
| August 2021 | 1676.6 | 1698.0 | 1.3% (-2.2% to 4.7%) |
| September 2021 | 1788.4 | 1847.1 | 3.3% (-0.08% to 6.7%) |
| October 2021 | 1877.1 | 1882.2 | 0.28% (-2.9% to 3.5%) |
| November 2021 | 1878.7 | 1981.2 | 5.5% (2.1% to 8.8%) |
| December 2021 | 1842.1 | 1917.9 | 4.1% (0.78% to 7.5%) |
| January 2022 | 1918.9 | 1930.3 | 0.61% (-2.6% to 3.8%) |
| February 2022 | 1799.0 | 1837.4 | 2.1% (-1.2% to 5.5%) |
| March 2022 | 1913.5 | 2061.6 | 7.7% (4.4% to 11.1%) |
| April 2022 | 1912.6 | 1961.9 | 2.6% (-0.68% to 5.8%) |
| May 2022 | 1972.8 | 2060.0 | 4.4% (1.2% to 7.7%) |
| June 2022 | 1845.3 | 2027.9 | 9.9% (6.4% to 13.4%) |

**Supplemental Table 7: Projected and Actual Stimulant Dispensing in Residents 0 to 24 years of age, Income Quintile 1, April 2020 to June 2022**

| **Month** | **Projected Rate of stimulant dispensing in absence of COVID pandemic (individuals per 100,000)** | **Actual Rate of stimulant dispensing during COVID pandemic (individuals per 100,000)** | **Relative percent change, actual versus projected stimulant dispensing (95% confidence interval** |
| --- | --- | --- | --- |
| April 2020 | 1431.9 | 1263.0 | -11.8% (-14.2% to -9.4%) |
| May 2020 | 1455.8 | 1304.0 | -10.4% (-12.8% to -8.0%) |
| June 2020 | 1362.3 | 1327.5 | -2.5% (-5.2% to 0.08%) |
| July 2020 | 1233.7 | 1259.3 | 2.1% (-0.77% to 4.9%) |
| August 2020 | 1259.7 | 1212.4 | -3.8% (-6.5% to -1.1%) |
| September 2020 | 1366.9 | 1310.8 | -4.1% (-6.7% to -1.5%) |
| October 2020 | 1428.8 | 168.8 | -4.2% (-6.7% to -1.7%) |
| November 2020 | 14451 | 1387.5 | -4.0% (-6.5% to -1.5%) |
| December 2020 | 1391.2 | 1357.1 | -2.4% (-5.0% to 0.15%) |
| January 2021 | 1477.8 | 1383.5 | -6.4% (-8.8% to -3.9%) |
| February 2022 | 1404.4 | 1356.1 | -3.4% (-6.0% to -0.85%) |
| March 2021 | 1485.9 | 1511.8 | 1.8% (-0.86% to 4.4%) |
| April 2021 | 1482.8 | 1395.7 | -5.9% (-8.3% to -3.4%) |
| May 2021 | 1507.4 | 1405.5 | -6.8% (-9.2% to -4.3%) |
| June 2021 | 1410.4 | 1354.5 | -4.0% (-6.5% to -1.4%) |
| July 2021 | 1277.2 | 1275.2 | -0.15% (-2.9% to 2.6%) |
| August 2021 | 1304.0 | 1304.0 | 0.00 % (-2.7% to 2.7%) |
| September 2021 | 1414.7 | 1419.9 | 0.37% (-2.3% to 3.0%) |
| October 2021 | 1478.7 | 1445.6 | -2.2% (-4.8% to 0.29%) |
| November 2021 | 1495.4 | 1509.6 | 0.96% (-1.6% to 3.5%) |
| December 2021 | 1439.5 | 1450.1 | 0.74% (-1.9% to 3.4%) |
| January 2022 | 1529.0 | 1468.5 | -3.9% (-6.4% to -1.5%) |
| February 2022 | 1452.9 | 1428.6 | -1.7% (-4.3% to 0.92%) |
| March 2022 | 1537.1 | 1582.9 | 3.0% (0.38% to 5.6%) |
| April 2022 | 1533.7 | 1527.3 | -0.41% (-3.0% to 2.1%) |
| May 2022 | 1559.0 | 1579.7 | 1.3% (-1.2% to 3.9%) |
| June 2022 | 1458.6 | 1580.7 | 8.4% (5.6% to 11.2%) |

**Supplemental Table 8: Projected and Actual Stimulant Dispensing in Residents 0 to 24 years of age, Income Quintile 2, April 2020 to June 2022**

| **Month** | **Projected Rate of stimulant dispensing in absence of COVID pandemic (individuals per 100,000)** | **Actual Rate of stimulant dispensing during COVID pandemic (individuals per 100,000)** | **Relative percent change, actual versus projected stimulant dispensing (95% confidence interval** |
| --- | --- | --- | --- |
| April 2020 | 1434.6 | 1272.4 | -11.3% (-13.7% to -8.9%) |
| May 2020 | 1463.6 | 1335.9 | -8.7% (-11.2% to -6.2%) |
| June 2020 | 1365.4 | 1365.7 | 0.03% (-2.7% to 2.8%) |
| July 2020 | 1224.5 | 1269.9 | 3.7% (0.75% to 6.7%) |
| August 2020 | 1260.9 | 1234.7 | -2.1% (-4.9% to 0.73%) |
| September 2020 | 1374.8 | 1339.3 | -2.6% (-5.3% to 0.09%) |
| October 2020 | 1440.1 | 1406.9 | -2.3% (-4.9% to 0.31%) |
| November 2020 | 1451.6 | 1423.9 | -1.9% (-4.5% to 0.72%) |
| December 2020 | 1405.3 | 1401.9 | -0.24% (-2.9% to 2.5%) |
| January 2021 | 1490.8 | 1410.8 | -5.4% (-7.9% to -2.8%) |
| February 2022 | 1407.6 | 1388.5 | -1.4% (-4.0% to 1.3%) |
| March 2021 | 1492.6 | 1548.3 | 3.7% (1.0% to 6.4%) |
| April 2021 | 1492.4 | 1435.8 | -3.8% (-6.3% to -1.2%) |
| May 2021 | 1522.4 | 1444.7 | -5.1% (-7.6% to -2.6%) |
| June 2021 | 14120.0 | 1406.7 | -0.92% (-3.6% to 1.7%) |
| July 2021 | 1273.3 | 1309.9 | 2.9% (-0.01% to 5.8%) |
| August 2021 | 1311.1 | 1339.0 | 2.1% (-0.71% to 5.0%)c |
| September 2021 | 1429.3 | 1474.5 | 3.2% (0.42% to 5.9%) |
| October 2021 | 1497.0 | 1501.6 | 0.31% (-2.3% to 2.9%) |
| November 2021 | 1508.7 | 1583.5 | 5.0% (2.3% to 7.7%) |
| December 2021 | 1460.4 | 1500.8 | 2.8% (0.06% to 5.5%) |
| January 2022 | 1549.1 | 1539.3 | -0.62% (-3.2% to 1.9%) |
| February 2022 | 1462.5 | 1493.4 | 2.1% (-0.58% to 4.8%) |
| March 2022 | 1550.5 | 1641.0 | 5.8% (3.1% to 8.5%) |
| April 2022 | 1550.2 | 1605.4 | 3.6% (0.90% to 6.2%) |
| May 2022 | 1581.1 | 1646.7 | 4.2% (1.5% to 6.8%) |
| June 2022 | 1474.6 | 1612.8 | 9.4% (6.5% to 12.2%) |

**Supplemental Table 9: Projected and Actual Stimulant Dispensing in Residents 0 to 24 years of age, Income Quintile 3, April 2020 to June 2022**

| **Month** | **Projected Rate of stimulant dispensing in absence of COVID pandemic (individuals per 100,000)** | **Actual Rate of stimulant dispensing during COVID pandemic (individuals per 100,000)** | **Relative percent change, actual versus projected stimulant dispensing (95% confidence interval** |
| --- | --- | --- | --- |
| April 2020 | 1321.2 | 1194.6 | -9.6% (-12.1% to -7.1%) |
| May 2020 | 1345.3 | 1254.0 | -6.8% (-9.3% to -4.3%) |
| June 2020 | 1253.8 | 1280.8 | 2.2% (-0.62% to 4.9%) |
| July 2020 | 1108.6 | 1183.4 | 6.7% (3.7% to 9.8%) |
| August 2020 | 1154.0 | 1145.4 | -0.74% (-3.6% to 2.1%) |
| September 2020 | 1275.0 | 1282.9 | 0.62% (-2.1% to 3.3%) |
| October 2020 | 1340.9 | 1329.1 | -0.88% (-3.5% to 1.7%) |
| November 2020 | 1354.2 | 1364.6 | 0.77% (-1.9% to 3.4%) |
| December 2020 | 1305.2 | 1338.4 | 2.5% (-0.19% to 5.3%) |
| January 2021 | 1373.1 | 1339.9 | -2.4% (-5.0% to 0.14%) |
| February 2022 | 1292.5 | 1295.7 | 0.25% (-2.4% to 2.9%) |
| March 2021 | 1375.6 | 1469.7 | 6.9% (4.1% to 9.6%) |
| April 2021 | 1372.2 | 1370.9 | -0.09% (-2.7% to 2.5%) |
| May 2021 | 1397.0 | 1356.7 | -2.9% (-5.4% to -0.35%) |
| June 2021 | 1301.9 | 1316.1 | 1.1% (-1.6% to 3.8%) |
| July 2021 | 1151.0 | 1223.1 | 6.3% (3.3% to 9.2%) |
| August 2021 | 1198.0 | 1265.2 | 5.6% (2.7% to 8.5%) |
| September 2021 | 1323.4 | 1399.7 | 5.8% (3.0% to 8.5%) |
| October 2021 | 1391.7 | 1428.1 | 2.6% (-0.02% to 5.3%) |
| November 2021 | 1405.3 | 1511.8 | 7.6% (4.9% to 10.3%) |
| December 2021 | 1354.4 | 1439.3 | 6.3% (3.5% to 9.0%) |
| January 2022 | 1424.6 | 1475.8 | 3.6% (0.96% to 6.2%) |
| February 2022 | 1340.8 | 1398.2 | 4.3% (1.6% to 7.0%) |
| March 2022 | 1426.9 | 1559.2 | 9.3% (6.5% to 12.0%) |
| April 2022 | 1423.3 | 1517.2 | 6.6% (3.9% to 9.3%) |
| May 2022 | 1448.8 | 1574.3 | 8.7% (5.9% to 11.4%) |
| June 2022 | 1350.0 | 1533.2 | 13.6% (10.6% to 16.5%) |

**Supplemental Table 10: Projected and Actual Stimulant Dispensing in Residents 0 to 24 years of age, Income Quintile 4, April 2020 to June 2022**

| **Month** | **Projected Rate of stimulant dispensing in absence of COVID pandemic (individuals per 100,000)** | **Actual Rate of stimulant dispensing during COVID pandemic (individuals per 100,000)** | **Relative percent change, actual versus projected stimulant dispensing (95% confidence interval** |
| --- | --- | --- | --- |
| April 2020 | 1391.4 | 1261.5 | -9.3% (-11.7% to -7.0%) |
| May 2020 | 1417.1 | 1307.5 | -7.7% (-10.1% to -5.4%) |
| June 2020 | 1316.7 | 1351.2 | 2.6% (-0.03% to 5.3%) |
| July 2020 | 1158.3 | 1242.6 | 7.3% (4.3% to 10.2%) |
| August 2020 | 1214.6 | 1216.9 | 0.20% (-2.5% to 2.9%) |
| September 2020 | 1341.9 | 1351.1 | 0.69% (-1.9% to 3.3%) |
| October 2020 | 1426.2 | 1425.7 | -0.03% (-2.5% to 2.5%) |
| November 2020 | 1436.9 | 1451.2 | 1.0% (-1.5% to 3.5%) |
| December 2020 | 1387.5 | 1413.3 | 1.9% (-0.71% to 4.4%) |
| January 2021 | 1454.7 | 1421.0 | 2.3% (-4.7% to 0.11%) |
| February 2022 | 1372.7 | 1380.0 | 0.53% (-2.0% to 3.1%) |
| March 2021 | 1452.5 | 1569.1 | 8.0% (5.4% to 10.7%) |
| April 2021 | 1457.5 | 1447.6 | -0.67% (-3.1% to 1.8%) |
| May 2021 | 1484.1 | 1439.4 | -3.0% (-5.4% to -0.62%) |
| June 2021 | 1378.8 | 1403.2 | 1.8% (-0.80% to 4.3%) |
| July 2021 | 1212.7 | 1279.1 | 5.5% (2.7% to 8.3%) |
| August 2021 | 1271.3 | 1322.5 | 4.0% (1.3% to 6.7%) |
| September 2021 | 1404.4 | 1463.4 | 4.2% (1.6% to 6.8%) |
| October 2021 | 1492.4 | 1524.0 | 2.1% (-0.36% to 4.6%) |
| November 2021 | 1503.3 | 1615.5 | 7.5% (4.9% to 10.0%) |
| December 2021 | 1451.4 | 1533.5 | 5.7% (3.1% to 8.2%) |
| January 2022 | 1521.5 | 1565.3 | 2.9% (0.42% to 5.4%) |
| February 2022 | 1435.4 | 1526.0 | 6.3% (3.7% to 8.9%) |
| March 2022 | 1518.6 | 1670.4 | 10.0% (7.4% to 12.6%) |
| April 2022 | 1523.6 | 1618.1 | 6.2% (3.7% to 8.8%) |
| May 2022 | 1551.2 | 1653.3 | 6.6% (4.1% to 9.1%) |
| June 2022 | 1440.8 | 1623.1 | 12.7% (9.9% to 15.4%) |

**Supplemental Table 11: Projected and Actual Stimulant Dispensing in Residents 0 to 24 years of age, Income Quintile 5, April 2020 to June 2022**

| **Month** | **Projected Rate of stimulant dispensing in absence of COVID pandemic (individuals per 100,000)** | **Actual Rate of stimulant dispensing during COVID pandemic (individuals per 100,000)** | **Relative percent change, actual versus projected stimulant dispensing (95% confidence interval** |
| --- | --- | --- | --- |
| April 2020 | 1578.5 | 1448.8 | -8.2% (-10.5% to -6.0%) |
| May 2020 | 1601.1 | 1507.5 | -5.8% (-8.1% to -3.6%) |
| June 2020 | 1474.7 | 1530.2 | 3.8% (1.2% to 6.3%) |
| July 2020 | 1277.2 | 1413.0 | 10.6% (7.8% to 13.5%) |
| August 2020 | 1370.8 | 1389.1 | 1.3% (-1.3% to 3.9%) |
| September 2020 | 1521.3 | 1584.2 | 4.1% (1.6% to 6.6%) |
| October 2020 | 1634.1 | 1671.3 | 2.3% (-0.11% to 4.7%) |
| November 2020 | 1653.8 | 1703.1 | 3.0% (0.59% to 5.4%) |
| December 2020 | 1586.0 | 1637.2 | 3.2% (0.79% to 5.7%) |
| January 2021 | 1673.6 | 1639.3 | -2.0% (-44.3% to 0.24%) |
| February 2022 | 1567.2 | 1607.9 | 2.6% (0.16% to 5.0%) |
| March 2021 | 1657.4 | 1803.5 | 8.8% (6.3% to 11.3%) |
| April 2021 | 1660.4 | 1659.1 | -0.07% (-2.4% to 2.3%) |
| May 2021 | 1683.7 | 1657.4 | -1.6% (-3.8% to 0.72%) |
| June 2021 | 1550.6 | 1601.0 | 3.3% (0.79% to 5.7%) |
| July 2021 | 1342.6 | 1445.1 | 7.6% (4.9% to 10.4%) |
| August 2021 | 1440.7 | 1531.5 | 6.3% (3.7% to 8.9%) |
| September 2021 | 1598.5 | 1724.4 | 7.9% (5.4% to 10.4%) |
| October 2021 | 1716.7 | 1782.0 | 3.8% (1.5% to 6.2%) |
| November 2021 | 1737.0 | 1894.5 | 9.1% (6.6% to 11.5%) |
| December 2021 | 1665.5 | 1777.9 | 6.8% (4.3% to 9.2%) |
| January 2022 | 1757.1 | 1863.3 | 6.1% (3.7% to 8.4%) |
| February 2022 | 1645.1 | 1768.9 | 7.5% (5.1% to 10.0%) |
| March 2022 | 1739.4 | 1967.4 | 13.1% (10.6% to 15.6%) |
| April 2022 | 1742.2 | 1868.1 | 7.2% (4.8% to 9.6%) |
| May 2022 | 1766.4 | 1960.0 | 11.0% (8.5% to 13.4%) |
| June 2022 | 1626.4 | 1916.1 | 17.8% (15.1% to 20.5%) |

**Supplemental Table 12: Projected and Actual Stimulant Dispensing in Children and Youth 5 to 9 of age, April 2020 to June 2022**

| **Month** | **Projected Rate of stimulant dispensing in absence of COVID pandemic (individuals per 100,000)** | **Actual Rate of stimulant dispensing during COVID pandemic (individuals per 100,000)** | **Relative percent change, actual versus projected stimulant dispensing (95% confidence interval** |
| --- | --- | --- | --- |
| April 2020 | 1771.6 | 1489.7 | -15.9% (-18.0% to -13.9%) |
| May 2020 | 1830.1 | 1519.0 | -17.0% (-19.0% to -15.0%) |
| June 2020 | 1697.1 | 1506.5 | -11.2% (-13.4% to -9.1%) |
| July 2020 | 1477.7 | 1390.2 | -5.9% (-8.3% to -3.5%) |
| August 2020 | 1511.9 | 1334.7 | -11.7% (-14.0% to -9.4%) |
| September 2020 | 1633.0 | 1426.2 | -12.7% (-14.9% to -10.5%) |
| October 2020 | 1731.2 | 1486.2 | -14.2% (-16.3% to -12.1%) |
| November 2020 | 1771.1 | 1509.8 | -14.8% (-16.8% to -12.7%) |
| December 2020 | 1707.9 | 1470.4 | -13.9% (-16.0% to -11.8%) |
| January 2021 | 1775.8 | 1458.3 | -17.9% (-19.9% to -15.9%) |
| February 2022 | 1707.2 | 1429.8 | -16.2% (-18.3% to -14.2%) |
| March 2021 | 1801.2 | 1602.4 | -11.0% (-13.2% to -8.9%) |
| April 2021 | 1826.3 | 1469.6 | -19.5% (-21.5% to -17.6%) |
| May 2021 | 1886.5 | 1486.4 | -21.2% (-23.1% to -19.3%) |
| June 2021 | 1749.2 | 1414.4 | -19.1% (-21.1% to -17.1%) |
| July 2021 | 1523.0 | 1247.4 | -18.1% (-20.2% to -15.9%) |
| August 2021 | 1558.1 | 1274.3 | -18.2% (-20.3% to -16.1%) |
| September 2021 | 1682.8 | 1372.0 | -18.5% (-20.5% to -16.4%) |
| October 2021 | 1783.8 | 1404.2 | -21.3% (-23.2% to -19.3%) |
| November 2021 | 1824.9 | 1467.3 | -19.6% (-21.5% to -17.6%) |
| December 2021 | 1759.6 | 1405.3 | -20.1% (-22.1% to -118.2%) |
| January 2022 | 1829.4 | 1405.2 | -23.2% (-25.1% to -21.3%) |
| February 2022 | 1758.6 | 1355.6 | -22.9% (-24.8% to -21.0%) |
| March 2022 | 1855.3 | 1487.1 | -19.8% (-21.8% to -17.9%) |
| April 2022 | 1881.0 | 1460.3 | -22.4% (-24.2% to -20.5%) |
| May 2022 | 1942.9 | 1518.6 | -21.8% (-23.7% to -20.0%) |
| June 2022 | 1801.4 | 1473.8 | -18.2% (-20.2% to -16.2%) |

**Supplemental Table 13: Projected and Actual Stimulant Dispensing in Children and Youth 10 to 14 years of age, April 2020 to June 2022**

| **Month** | **Projected Rate of stimulant dispensing in absence of COVID pandemic (individuals per 100,000)** | **Actual Rate of stimulant dispensing during COVID pandemic (individuals per 100,000)** | **Relative percent change, actual versus projected stimulant dispensing (95% confidence interval** |
| --- | --- | --- | --- |
| April 2020 | 2406.7 | 2193.3 | -8.9% (-10.7% to -7.0%) |
| May 2020 | 2488.7 | 2289.8 | -8.0% (-9.8% to -6.2%) |
| June 2020 | 2269.6 | 2301.9 | 1.4% (-0.62% to 3.5%) |
| July 2020 | 1950.9 | 2091.4 | 7.2% (4.9% to 9.5%) |
| August 2020 | 2018.3 | 2056.8 | 1.9% (-0.27% to 4.1%) |
| September 2020 | 2299.5 | 2325.9 | 1.1% (-0.88% to 3.2%) |
| October 2020 | 2424.1 | 2449.6 | 1.1% (-0.92% to 3.0%) |
| November 2020 | 2440.8 | 2526.5 | 3.5% (1.5% to 5.5%) |
| December 2020 | 2356.5 | 2430.5 | 3.1% (1.1% to 5.2%) |
| January 2021 | 2471.7 | 2409.9 | -2.5% (-4.4% to -0.60%) |
| February 2022 | 2327.8 | 2360.0 | 1.4% (-0.63% to 3.4%) |
| March 2021 | 2443.2 | 2652.9 | 8.6% (6.5% to 10.7%) |
| April 2021 | 2455.2 | 2372.8 | -3.4% (-5.2% to -1.5%) |
| May 2021 | 2538.7 | 2392.8 | -5.7% (-7.6% to -3.9%) |
| June 2021 | 2315.2 | 2270.0 | -1.9% (-3.9% to 0.02%) |
| July 2021 | 1990.1 | 2031.2 | 2.1% (-0.11% to 4.3%) |
| August 2021 | 2058.7 | 2106.1 | 2.3% (0.16% to 4.5%) |
| September 2021 | 2345.5 | 2434.8 | 3.8% (1.8% to 5.9%) |
| October 2021 | 2472.5 | 2507.0 | 1.4% (-0.55% to 3.4%) |
| November 2021 | 2489.5 | 2650.8 | 6.5% (4.5% to 8.5%) |
| December 2021 | 2403.4 | 2469.6 | 2.8% (0.76% to 4.7%) |
| January 2022 | 2520.8 | 2503.3 | -0.69% (-2.6% to 1.2%) |
| February 2022 | 2373.9 | 2425.0 | 2.2% (0.15% to 4.2%) |
| March 2022 | 2491.5 | 2644.7 | 6.1% (4.1% to 8.2%) |
| April 2022 | 2503.7 | 2560.4 | 2.3% (0.32% to 4.2%) |
| May 2022 | 2588.8 | 2682.4 | 3.6% (1.7% to 5.6%) |
| June 2022 | 2360.8 | 2555.7 | 8.3% (6.2% to 10.4%) |

**Supplemental Table 14: Projected and Actual Stimulant Dispensing in Children and Youth 15 to 19 years of age, April 2020 to June 2022**

| **Month** | **Projected Rate of stimulant dispensing in absence of COVID pandemic (individuals per 100,000)** | **Actual Rate of stimulant dispensing during COVID pandemic (individuals per 100,000)** | **Relative percent change, actual versus projected stimulant dispensing (95% confidence interval** |
| --- | --- | --- | --- |
| April 2020 | 1684.1 | 1488.2 | -11.6% (-13.8% to -9.5%) |
| May 2020 | 1707.6 | 1547.0 | -9.4% (-11.6% to -7.2%) |
| June 2020 | 1595.6 | 1579.4 | -1.0% (-3.4% to 1.4%) |
| July 2020 | 1390.4 | 1464.3 | 5.3% (2.6% to 8.0%) |
| August 2020 | 1486.1 | 1443.0 | -2.9% (-5.4% to -0.44%) |
| September 2020 | 1657.5 | 1636.3 | -1.3% (-3.6% to 1.1%) |
| October 2020 | 1749.1 | 1734.1 | -0.85% (-3.1% to 1.4%) |
| November 2020 | 1748.7 | 1734.6 | -0.81% (-3.1% to 1.5%) |
| December 2020 | 1684.8 | 1689.9 | 0.31% (-2.0% to 2.7%) |
| January 2021 | 1789.9 | 1720.2 | -3.9% (-6.1% to -1.7%) |
| February 2022 | 1670.6 | 1658.1 | -0.74% (-3.1% to 1.6%) |
| March 2021 | 1755.0 | 1876.1 | 6.9% (4.5% to 9.3%) |
| April 2021 | 1773.1 | 1744.9 | -1.6% (-3.9% to 0.68%) |
| May 2021 | 1797.5 | 1743.8 | -3.0% (-5.2% to -0.75%) |
| June 2021 | 1679.2 | 1702.0 | 1.4% (-1.0% to 3.7%) |
| July 2021 | 1463.0 | 1557.3 | 6.5% (3.8% to 9.1%) |
| August 2021 | 1563.3 | 1635.8 | 4.6% (2.1% to 7.2%) |
| September 2021 | 1743.3 | 1831.6 | 5.1% (2.7% to 7.5%) |
| October 2021 | 1839.2 | 1881.3 | 2.3% (0.01% to 4.6%) |
| November 2021 | 1838.4 | 1988.6 | 8.2% (5.8% to 10.6%) |
| December 2021 | 1770.9 | 1870.5 | 5.6% (3.2% to 8.0%) |
| January 2022 | 1881.0 | 1938.3 | 3.1% (0.78% to 5.3%) |
| February 2022 | 1755.3 | 1867.0 | 6.4% (4.0% to 8.8%) |
| March 2022 | 1843.5 | 2048.0 | 11.1% (8.7% to 13.5%) |
| April 2022 | 1862.2 | 1966.5 | 5.6% (3.3% to 7.9%) |
| May 2022 | 1887.4 | 2039.2 | 8.0% (5.7% to 10.4%) |
| June 2022 | 1762.9 | 1982.4 | 12.5% (10.0% to 15.0%) |

**Supplemental Table 15: Projected and Actual Stimulant Dispensing in Children and Youth 20 to 24 years of age, May 2020 to June 2022**

| **Month** | **Projected Rate of stimulant dispensing in absence of COVID pandemic (individuals per 100,000)** | **Actual Rate of stimulant dispensing during COVID pandemic (individuals per 100,000)** | **Relative percent change, actual versus projected stimulant dispensing (95% confidence interval** |
| --- | --- | --- | --- |
| May 2020 | 1082.8 | 1188.1 | 9.7% (6.7% to 12.7%) |
| June 2020 | 1052.7 | 1288.7 | 22.4% (19.1% to 25.7%) |
| July 2020 | 1057.5 | 1247.8 | 18.0% (14.8% to 21.2%) |
| August 2020 | 1115.9 | 1195.0 | 7.1% (4.2% to 10.0%) |
| September 2020 | 1134.7 | 1302.2 | 14.8% (11.7% to 17.8%) |
| October 2020 | 1198.7 | 1343.4 | 12.1% (9.2% to 15.0%) |
| November 2020 | 1209.9 | 1364.4 | 12.8% (9.9% to 15.7%) |
| December 2020 | 1155.6 | 1358.2 | 17.5% (14.5% to 20.6%) |
| January 2021 | 1262.6 | 1401.4 | 11.0% (8.2% to 13.8%) |
| February 2022 | 1169.2 | 1375.5 | 17.7% (14.6% to 20.7%) |
| March 2021 | 1284.1 | 1538.7 | 19.8% (16.9% to 22.8%) |
| April 2021 | 1233.7 | 1497.5 | 21.4% (18.4% to 24.4%) |
| May 2021 | 1170.5 | 1456.3 | 24.4% (21.2% to 27.6%) |
| June 2021 | 1137.4 | 1469.8 | 29.2% (25.9% to 32.6%) |
| July 2021 | 1142.1 | 1470.5 | 28.8% (25.5% to 32.1%) |
| August 2021 | 1204.6 | 1513.4 | 25.6% (22.5% to 28.8%) |
| September 2021 | 1224.3 | 1595.3 | 30.3% (27.1% to 33.5%) |
| October 2021 | 1292.7 | 1635.7 | 26.5% (23.5% to 29.6%) |
| November 2021 | 1304.1 | 1738.0 | 33.3% (30.1% to 36.5%) |
| December 2021 | 1245.0 | 1690.3 | 35.8% (32.5% to 39.1%) |
| January 2022 | 1359.7 | 1787.6 | 31.5% (28.4% to 34.6%) |
| February 2022 | 1258.6 | 1680.0 | 33.5% (30.2% to 36.7%) |
| March 2022 | 1381.6 | 1896.1 | 37.2% (34.1% to 40.4%) |
| April 2022 | 1326.7 | 1793.1 | 35.2% (31.9% to 38.4%) |
| May 2022 | 1258.3 | 1787.6 | 42.1% (38.6% to 45.5%) |
| June 2022 | 1222.2 | 1841.9 | 50.7% (47.0% to 54.4%) |

Supplemental Figure 1: Impact of COVID-19 (April 2020) on monthly rates of stimulant dispensing among Ontario residents between the ages of 0 and 4, January 2013 to June 2022


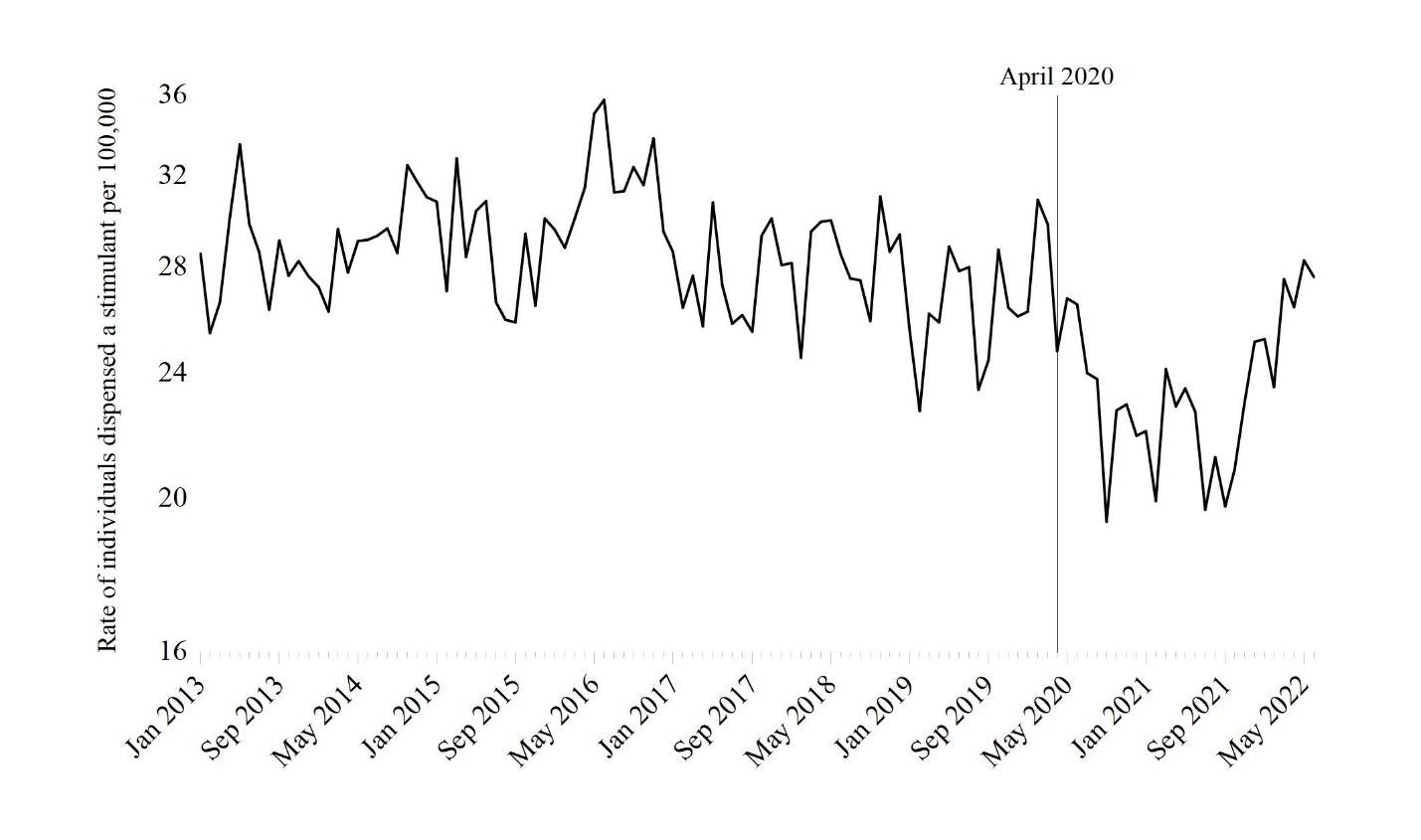

Supplement: Supplementary file 1 — (DOCX 197 KB) [file 787_2023_2346_MOESM1_ESM.docx]
